# A Novel Antireflux Technique for Orthotopic Ileal Bladder Substitutes—Flat-Segment Technique: Preliminary Results

**DOI:** 10.5402/2011/431951

**Published:** 2011-09-14

**Authors:** Hany ElFayoumy, Ashraf Abou-Elela, Tamer Orban, Ashraf Emran, Mohamed Elghoneimy, Ahmed Morsy

**Affiliations:** Urology Department, Kasr Al Aini Hospitals, Cairo University, Cairo, Egypt

## Abstract

*Objective*. Although a large debate exists regarding the need for reflux prevention in ileal orthotopic neobladders, it is our policy to continue performing nonrefluxing ureteroileal anastomoses for our patients. An ideal uretero-ileal anastomosis must be simple, nonrefluxing, as well as non-obstructive. Here, we present a new antireflux mechanism for orthotopic ileal neobladders. *Methods*. 12 radical cystectomy patients for muscle invasive bladder cancer were candidates for orthotopic urinary diversion and underwent a non-refluxing uretero-ileal anastomosis using the flat-segment technique with a follow up of 6 to 18 months. *Results*. Preliminary results after the short-term followup showed that the success rate in reflux prevention was 92% and no cases of obstruction. The upper tracts were preserved or improved in all 12 patients. Operative time for neobladder creation ranged between 120–240 minutes, with a mean of 165 minutes (±36 minutes). No diversion-related complications. *Conclusions*. Based on our early data, we believe that the flat-segment uretero-ileal anastomosis technique for reflux prevention in orthotopic ileal bladder substitutes is simple, easy to learn and carries no additional morbidity to a standard refluxing uretero-ileal anastomosis, but has the advantage of effective reflux prevention. A longer follow-up period study with more patient numbers is ongoing.

## 1. Introduction

For more than a century the intestinal segments have been used in reconstruction of the lower urinary tract. The developments in the last two decades in the field of continent urinary diversion and bladder substitution have made these operations a standard treatment of irreversibly damaged or diseased bladder. The ideal form of continent urinary reservoirs should be low pressure, highly compliant, of adequate capacity, should maintain continence, and preserve the integrity of upper tract through a nonobstructing antireflux mechanism and should empty Completely [1, 2].

## 2. Materials and Methods

Twelve consecutive patients were included in this prospective study. A detailed signed consent was obtained.

All 12 patients underwent radical cystectomy for invasive carcinoma of the bladder followed by ileal bladder substitute

Inclusion criteria included (A) patients with serum creatinine below 2 mg/dL; (B) patients who have no evidence of tumor encroaching on the bladder neck or prostatic urethra; (C) patients with good hepatic and cardiopulmonary functions; (D) patients who are motivated with a good performance status, understanding the nature of disease, and are willing to adhere to a proper follow-up regimen. 

The diameter of the ureters did not affect decision making regarding the construction of the pouch. 

**Figure 1:**
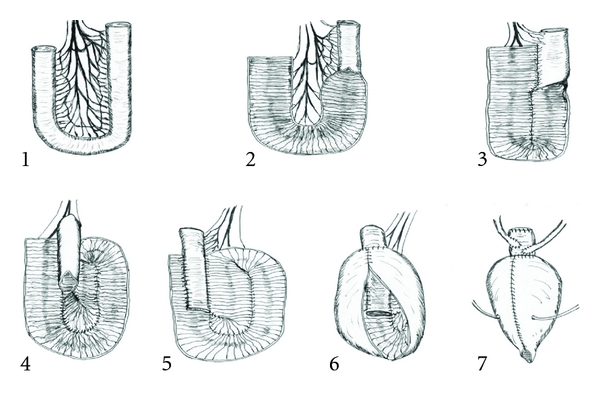
Steps for creation of the bladder substitute with the flat-segment valve technique.

After radical cystoprostatectomy was completed, the bowel segment was selected from the distal ileum, sparing one foot proximal to the ileo-cecal valve. The bowel segment ranged between 40–45 cm. (Figure 1, step 1). Intestinal continuity was restored. The distal 35 cm of the isolated segment was detubularized (Figure 1, step 2). The opened ileum and the adjacent proximal 10 cm of the intact bowel (valvular segment) were used to fashion a double folded ileal pouch. 

A U-shaped ileal fold was created by suturing the opened ileal plate to the adjacent border of the valvular segment along the junction between the mesentery and the serosal surface using continuous 3-0 polyglycolic acid suture. At the distal end of the valve, the opened adjacent edges were sutured together to complete the first fold (Figure 1, step 3).

The caudal opened ileum was rotated upwards to be adjacent to the valvular segment, and the mucosal edge was again sutured to the valvular segment at its mesenteric border as done previously on the other side (Figure 1, step 4). 

The antimesenteric border of the 7 cm valvular segment is then sutured to the middle of the floor of the adjacent mucosa of the opened ileum, as shown in Figure 1, step 5; with continuous 3-0 polyglycolic acid sutures, taking serosa and musculosa of the valvular segment and the mucosa and musculosa of the opened ileum.

The pouch was then closed by suturing the mucosal edges as shown in Figure 1 step 6.

Both ureters were anastomosed end to side to the supravalvular segment using interrupted 4-0 polyglycolic acid sutures. Ureters were anastomosed together in a Wallace fashion in cases were the ureters were dilated then anastomosed to the open oral end of the supravalvular segment. Ureteral stents (6 Fr.) are introduced into the ureters. The cephalic end of the supravalvular segment was closed using continuous 3-0 polyglycolic acid suture (Figure 1, step 7).

**Figure 2:**
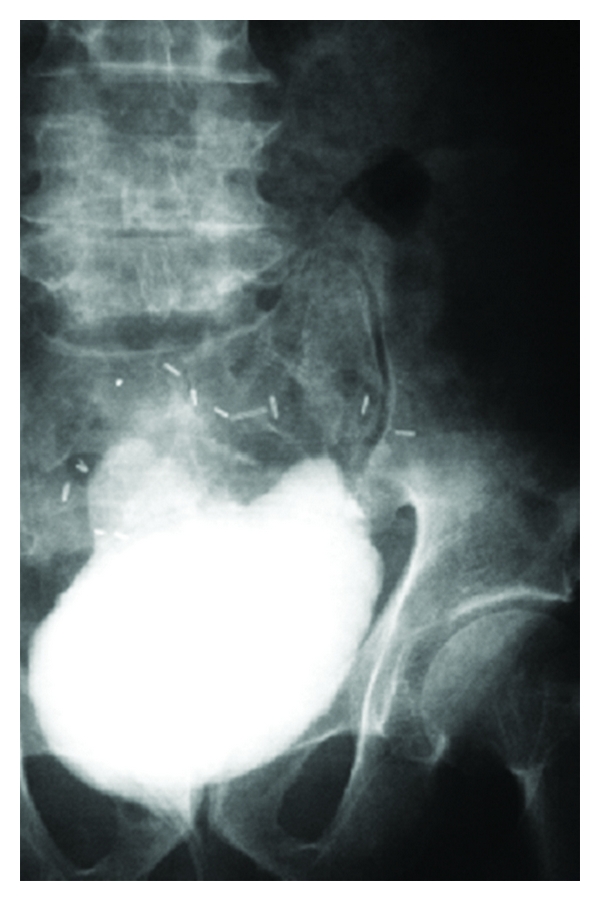


**Figure 3:**
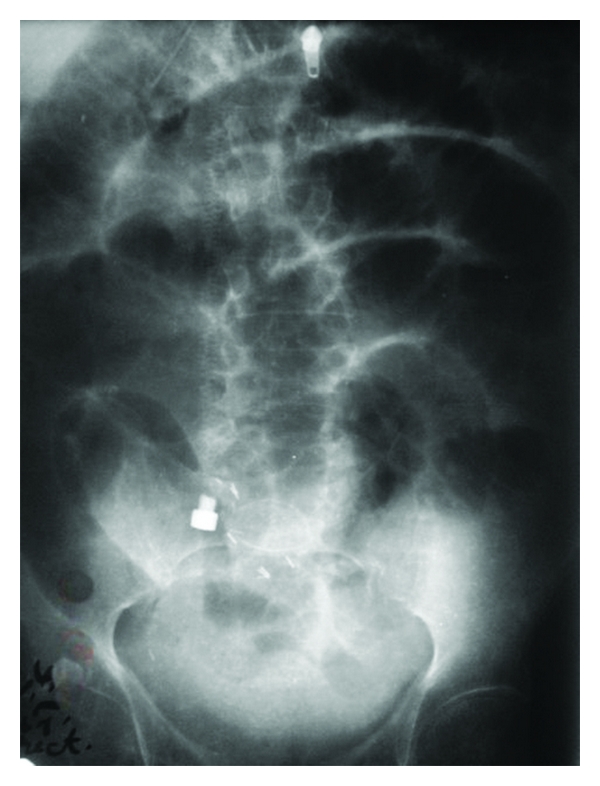


Patients were followed up for a minimum of 6 months to evaluate the efficacy of the valve. Most patients were followed up for more than 1 year.,

Follow-up regimen included questioning the patient about continence state. Complete continence was defined as complete urine control without the need for any protective pads by day. Lab evaluation included serum creatinine, blood urea, Na, K., arterial blood gases, urine culture, and alkaline phosphatase levels. Other labs were performed according to the need and indications: abdominal and pelvic ultrasound was performed with each follow-up visit to assess the condition of the kidneys and emptying of the pouch. Pouchogram was performed at 3 weeks to assess the healing of the pouch and to confirm the absence of extravasations (Figure 2). Pouchogram was also obtained at 3 months postoperatively to evaluate the anti-reflux mechanism and to assess the maturity of the pouch as regards capacity and the presence of residual urine (Figure 3).

**Figure 4:**
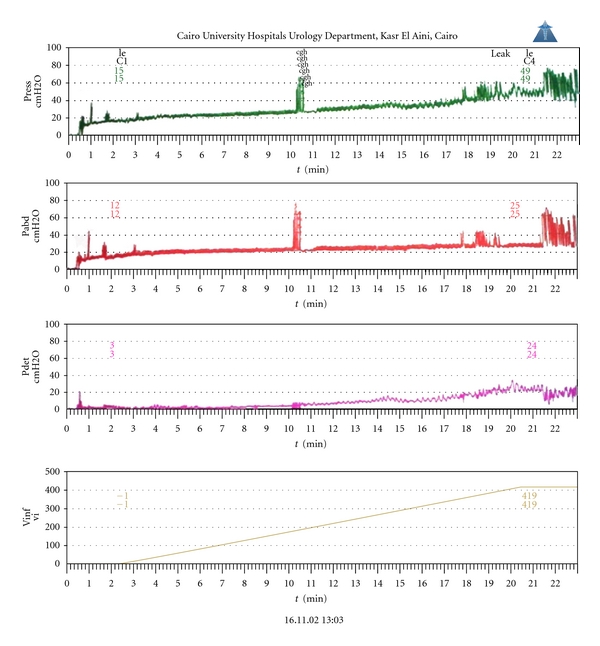


**Figure 5:**
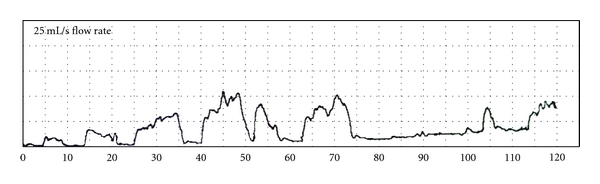


Urodynamic evaluation was done at 6 months postoperatively in all patients including uroflowmetry and cystometry (Figures 4 and 5). 

## 3. Results

The age ranged between 59 and 70 years with a mean of 63.2 ± 3.3 years. All patients were males. The indication for surgery was invasive bladder cancer in all 12 patients. The pathology was transitional cell carcinoma in 7 patients (58.3%) and squamous cell carcinoma in 5 patients (41.7%).

The operative time for pouch creation was defined as the time elapsing from the start of bowel loop selection till the beginning of wound closure and ranged between 120 minutes and 240 minutes, with a mean of 165 minutes ±36 minutes.

At a follow-up period of 6 months, no cases of obstruction were met. Eleven patients had no reflux (92%). The remaining patient had bilateral reflux. During cystoscopy, the flat segment was found to be cylindrical rather than flat as the row of sutures that fixes the segment to the floor of the pouch seemed disrupted.

Eleven patients were completely dry by day (91.7%). Of these patients some patient had occasional nighttime spotting requiring protective pads only. Eight patients (66.7%) had efficient emptying of the pouch using abdominal straining and Crédè's method. Four Patients (33.3%) had residual urine over 100 c.c. requiring clean intermittent catheterization. One of these patients had a large pouch capacity on a pouchogram measuring over 700 c.c. at sensation of fullness. The other 3 patients had a pouch capacity at sensation of fullness ranging between 400–700 c.c.

One patient had incontinence by day and night. This patient had a good pouch capacity on pouchogram ranging around 400 c.c. In this patient, there was excessive dissection around the prostatic apex to control bleeding. The patient was satisfied wearing a condom catheter and refused further operative procedures.

Uroflowmetry was performed in all patients. The maximum flow rate ranged between 11–38 mL/sec. with a mean of 27.3 mL/sec. ± 6.4 mL/sec. The average flow rate ranged between 3–14 mL/sec with a mean of 6.4 mL/sec ± 3.7 mL/sec. The voiding pattern was essentially a straining pattern in all patients. The voided volume ranged between 362–978 mL with a mean of 587.4 mL ± 149.2 mL.

Cystometric evaluation was performed in 10 nonrefluxing patients at 6 months post-operatively. The pouch capacity at sensation of fullness ranged between 420–1100 c.c. with a mean of 689.9 c.c. ± 195.9 c.c. The pouch pressure at sensation of fullness ranged between 15–39 cm H_2_O with a mean of 24 cm H_2_O ± 7 cm H_2_O. No high pressure spikes were noted in the filling phase. During the voiding phase, high pressure spikes reaching high above 100 cm H_2_O were noted in 7 patients. These high pressure spikes were primarily caused by abdominal straining and Crédè's method required for efficient emptying. Static urethral pressure profilometry was done in the 10 patients showing sphincteric activity within normal.

## 4. Discussion

A controversy exists regarding the need to perform an antireflux mechanism in ureteroileal anastomosis in patients undergoing orthotopic urinary diversion.

Those against reflux prevention suggest that, as a result of bowel detubularization and reconfiguration, the neobladder should accommodate a large volume of urine at low intrareservoir pressures [20]. In one study, Gotoh and associates, investigated the urodynamic parameters and pouch-urethral function involved in the storage and evacuation of urine in 18 patients undergoing an orthotopic ileal substitute. They provided urodynamic evidence that voiding is performed by increasing intraabdominal pressure. This Valsalva's maneuver, however, significantly increased intrareservoir pressures, with 44% of patients demonstrating considerably higher pressures (80 to 150 cm H_2_O) during voiding [21]. Another study by Shaaban et al. emphasized the advantages of non-refluxing ureterointestinal anastomosis in orthotopic bladder substitution, based on their experience of persistent bacteriuria in about one-third of their patients and mean pressures of 77.3 cm H_2_O during reservoir emptying with potential spread of microorganisms into the upper urinary tract [22].

Complete emptying of the neobladder is routinely performed with Valsalva's maneuver (increased abdominal pressure) and simultaneous relaxation of the external sphincter. In this situation, intra-abdominal pressure theoretically affects the neobladder, ureters, and renal pelvis. This concomitantly directed pressure prevents urinary reflux and protects the upper urinary tract [23]. Based on Studer's argument to prevent reflux in those patients with an isoperistaltic afferent limb, the intra-abdominal pressure on the ureter and the renal pelvis must be similar to that on the reservoir. Force, however, is a function of pressure applied to the surface area (force = pressure × surface area). Because the force on the neobladder is larger (a result of a larger surface area) than that on either the ureter or the renal pelvis, it is likely that reflux exists during these higher voiding pressure phases [21].

The need for clean intermittent catheterization is another confounding factor in this debate. Steven and Poulsen reported 34% 3-year and 24% 5-year prevalence of bacteriuria in 166 men undergoing orthotopic reconstruction. Furthermore, patients may unexpectedly require some form of intermittent catheterization to completely empty the neobladder which cannot be predicted preoperatively. Patients requiring catheterization will clearly be exposed to colonized bacteriuria. It has been reported that as many as 30% of women and 44% of men undergoing orthotopic diversion ultimately require some form of intermittent catheterization [24].

Stamey evaluated the long-term effects of urinary tract infections in adults and concluded that the adult kidney is highly resistant to renal damage by bacteria in the absence of obstruction and even in patients with progressive renal failure the presence of bacteria was not responsible for progression. Most of the changes of chronic pyelonephritis are seen in infancy, probably because the growing kidney is most susceptible to scarring which does not seem to occur in adults [25].

In animal models, a causative relationship between intra-renal reflux (IRR) and renal scarring has been confirmed. Two types of renal papillae have been described: a convex papilla in which the collecting ducts open obliquely on the papillary surface, preventing reflux and a concave or flat papilla, where the collecting ducts open at right angles and into which reflux can occur. In the humans the concave or flat papillae are located mainly in the polar regions of the kidney but are also found in the midzone of the kidney. The theory proposes that IRR occurs at the papillae as soon as the pressure gradient between the calyx and the collecting tubules is reversed (>10–15 mmHg). A prerequisite for scar formation in the kidney is the presence of concave papillae, and the theory explains why scars never develop in some kidneys despite reflux [26]. Obviously the predominant type of papillae cannot be predicted preoperatively.

Several methods of uretero-ileal implantation have been presented with different rates of success. In Table 1 we present the results of different types of uretero-ileal anastomosis commonly used by urologists.

The theoretical benefit of reflux prevention is lost if the ureteral implantation technique has a high rate of stricture formation. The ideal ureteroenteric anastomosis would be easy to construct and have a low incidence of stenosis. In general, the literature supports the notion that the risk of ureteral obstruction after a non-refluxing anastomosis is approximately twice that following a direct anastomosis [25].

In our series, the uretero-ileal anastomosis was performed in a simple direct end-to-side fashion to the chimney. The anti-reflux mechanism was done in the ileal valve segment below the chimney. In this way, we achieve an anti-reflux mechanism that has the same incidence of stenosis as the direct implantation methods.

The wider lumen and relatively thick wall of the ileum in comparison to the ureter explain why stenosis is unlikely to occur in the valve segment.

In this way, we have an efficient anti-reflux mechanism 92% (one refluxing patient out of 12 (8%)) with the same expected percent of stenosis as a direct anastomosis (0% in our series). These results are comparable to the most popular anti-reflux methods mentioned above. A study with more patient numbers and longer followup is underway.

## 5. Conclusions

Based on our early data, we believe that the flat-segment uretero-ileal anastomosis technique for reflux prevention in orthotopic ileal bladder substitutes is simple, easy to learn and carries no additional morbidity to a standard refluxing uretero-ileal anastomosis, but has the advantage of effective reflux prevention. The technique deserves further evaluation through larger number of patients and longer periods of followup.

## Figures and Tables

**Table 1:** 

Procedure	Renal units	Stenosis	Reflux
Le-Duc:			
Le-Duc et al. [3]	260	2%	15%
Shaaban et al. [4]	38	29%	2.6%

Serous lined extramural tunnel:			
Abol-Enein and Ghoneim [5]	102	3%	0%
Papadopoulos and Jacobsen [6]	50	4%	2%
Osman et al. [7]	39	5.1%	7.7%

Split cuff technique (nipple):			
Stone and MacDermott [8]	36	0%	3%
Sagalowsky [9]	98	3.1%	4.2%

Subserosal tunneling:			
Starr et al. [10]	20	10%	0%

Serosal fixation an astomosis:			
Itatani and Sonoda [11]	20	0%	15%

Hammock anastomosis:			
Hirdes et al. [12]	57	6%	20%

Intussuscepted valve (Kock):			
Elmajian et al. [13]	295	1.4%	2%
Osman et al. [7]	38	5.25%	5.25%

Antireflux hydraulic valve:			
Benchekroun [14]	210	20%	0%

Afferent isoperistaltic ileal segment:			
Studer et al. [15]	40	4%	0%
Studer et al. [16]	70	35%	0%
Courteny et al. [17]	100	6%	0%

Wall-incorporated anti-reflux valve:			
El-Bakry [18]	34	0.03%	0%

T-pouch:			
Stein et al. [19]	209	7.2%	10%
